# Mesoporous silica SBA-15 decreases hyperammonemia and affects the gene expression of mitogen-activated protein kinases in the prefrontal cortex of rats with bile duct ligation 

**DOI:** 10.22038/ijbms.2020.44658.10436

**Published:** 2020-10

**Authors:** Shamseddin Ahmadi, Halaleh Ghaderi, Nazila Saadati, Saadi Samadi

**Affiliations:** 1 Department of Biological Science, Faculty of Science, University of Kurdistan, Sanandaj, Iran; 2 Department of Chemistry, Faculty of Science, University of Kurdistan, Sanandaj, Iran

**Keywords:** Ammonia, c-Jun Kinase 3, Hepatic encephalopathy, Inflammations, p38 MAPK, SBA-15

## Abstract

**Objective(s):**

We aim to examine possible ammonia lowering effects of mesoporous silica SBA-15 in rats after the common bile duct ligation (BDL). We also evaluate the effect of SBA-15 treatments during 28 days of BDL on locomotion and rearing behavior, as well as on the gene expression of Jnk3 and p38alpha (p38α) mitogen-activated protein kinases in the prefrontal cortex (PFC).

**Materials and Methods:**

SBA-15 was prepared with the hydrothermal method from the surfactant P123 and tetraethyl orthosilicate (TEOS), and calcined at 550 ºC. Then, the product was characterized by FT-IR, XRD, SEM, and BJH-BET techniques. Male Wistar rats in sham control and a group with BDL received saline but another group with BDL received SBA-15 during 28 days of BDL. We examined all groups of rats weekly for locomotion and rearing behavior; then on day 28, we sacrificed all rats, collected the blood sample, and finally dissected the PFC from the whole brain.

**Results:**

The SBA-15 treatments had no effect on locomotion but improved rearing behavior on days 7 and 14 of BDL. Biochemical analysis indicated that the SBA-15 treatments in rats with BDL significantly decreased hyperammonemia. The results also revealed that the SBA-15 treatments in rats with BDL significantly restored the decreased Jnk3 gene expression, and increased the p38α gene expression in the PFC.

**Conclusion:**

We conclude that SBA-15 can be used as an ammonia lowering agent in hepatic encephalopathy; however, its improving effects on locomotion and neuroinflammation, as well as signaling molecules in the brain need more investigations.

## Introduction

Ammonia is produced during the metabolism of nitrogenous compounds such as amino acids and organic bases, as well as by ammonia-producing bacteria in the gastrointestinal tract (1). The liver acts as a central organ in detoxifying ammonia by its conversion to urea via the urea cycle (2, 3). Liver dysfunctions and the insufficient removal of ammonia from blood lead to systemic hyperammonemia, which in turn increases the possibility of the elevated ammonia level in other organs such as the brain (4, 5). Hyperammonemia in the brain causes a condition known as hepatic encephalopathy (HE) with complex neurological and neuropsychological outcomes (5, 6). Increased level of ammonia also activates microglia and production of cytokines, which both can induce neuroinflammation with some adverse outcomes in HE (7-9). 

Because of the pivotal role of ammonia in the induction of HE, therapeutic strategies in the treatment of the disease have focused on either prevention of excess ammonia production or its removal from the body (2, 3, 10, 11). However, the available therapeutic options are often unsatisfactory, and sometimes with undesired side effects (1, 12). Therefore, introducing innovative and effective agents for controlling the amount of ammonia production and/or decreasing the hyperammonemia in HE is of great interest (13). Mesoporous silica nanoparticles because of their controllable shape and high biocompatibility are inorganic delivery systems that are considered as appropriate models for drug delivery in the area of biomedicine (14, 15). One of the best-known mesoporous silicates is Santa Barbara Amorphous No. 15 (SBA-15) (16). SBA-15 has been widely used as a special carrier for vaccination and therapeutic purposes in cancer therapy (17-19). According to our knowledge, mesoporous silica SBA-15 has not ever been used as a drug carrier in the treatment of HE model animals. 

In rodents, exposure to an excess amount of ammonia (by infusion of an ammonium salt) rapidly increases the ammonia concentration that in turn leads to HE. Ligation of a common bile duct in rodents can also induce chronic liver failure, which is usually accompanied by hyperammonemia to develop an animal model of HE (7, 20, 21). According to findings in the field of HE, signaling pathways including mitogen-activated protein kinases (MAPKs) have a main role in the pathogenesis and treatment of HE (22, 23). In particular, it has been indicated that inhibition of MAPKs improves the cognitive impairments in HE models animals (4, 24). There is evidence indicating that p38α and c-Jun N- terminal kinase (JNK) inhibitors can prevent the synthesis of pro-inflammatory cytokines (22, 25-27). Besides, activation of p38 and JNK MAPKs in cultured astrocytes causes the astrocyte swelling and inhibits glutamate uptake (28-30). Therefore, a possible mechanism of action for MAPKs in HE may be mediated via induction of neuroinflammation and astrocyte swelling. 

A growing body of evidence confirms that hypo-locomotion and impairments in cognitive functions are major complications of HE, which is mainly induced by hyperammonemia (4, 31-33). Considering the porous shape of SBA-15, it may adsorb the increased ammonia levels in situations like HE. We aimed to evaluate a possible decreasing role of SBA-15 on hyperammonemia, and subsequent consequence on locomotion and rearing behavior in HE model rats with ligation of the common bile duct. In the second part of our study, we studied possible changes in the gene expression of Jnk3 and p38α MAPKs in the prefrontal cortex (PFC) to elucidate the impact of SBA-15 on these signaling molecules downstream to inflammatory cytokine and neurotransmitter receptors.

## Materials and Methods

***Animals***

Twenty-four male Wistar rats weighing 300-350 g were used (Pasteur Institute, Tehran, Iran). We kept the animals in a colony room under standard conditions (22±2 ^°^C, and a 12 hr light/dark cycle, which light on at 7:00 AM). We ensured the *ad libitum* access of the animals to food and water except during a fasting period before a surgical laparotomy and also during behavioral testing. The animals were randomly assigned to one of three experimental groups. The behavioral tests were carried out in a room with light and sound attenuation (between 9:00 and 12:00 AM). All procedures were performed under the National Institutes of Health (NIH) Guide for the Care and Use of Laboratory Animals (NIH Publications, No. 80–23, revised 1996).

***Surgical laparotomy***

We restricted the access of the animals to food but not water 12 hr before the surgery. Then, the animals were moved to the laboratory and left for 30 min under light and sound attenuated condition. Each rat underwent laparotomy under general anesthesia induced by the intraperitoneal (IP) administration of ketamine plus xylazine (100 + 10 mg/kg, respectively). The rats in a sham control group experienced laparotomy but without bile duct ligation (BDL) and resection. The rats in the intervention group were subjected to laparotomy and BDL according to our previous reports (34, 35). After completion of the laparotomy, each rat received 1 ml saline (IP), and left in a separate box for two additional hours to recover from the surgery. 

***Synthesis and analysis of mesoporous silica SBA-15***

Mesoporous silica SBA-15 was synthesized by a method described in the literature (36, 37). In a round bottom flask (500 ml), 12.0 g of Pluronic P123 (EO20PO70EO20) as a surfactant was dissolved in 275 mL ddH2O and 60 ml of HCl (37%) at room temperature. After complete copolymer dissolution (0.5-1 hr), 27.4 g of tetraethyl orthosilicate (TEOS) as a silicium (Si) source in ddH_2_O (100 ml) was added and stirred vigorously at 40 ^°^C for 30 min. Next, the resulting mixture was transferred to a stainless steel jacketed teflon vessel and heated at 100 ^°^C for 48 hr. After cooling the mixture of the reaction to room temperature, the solid product was separated by filtration and then washed by ddH_2_O until pH=7-8 achieved. Finally, the obtained solid product was dried at 60 ^°^C, and the obtained powder calcined at 550 ^°^C for 5 hr.

Fourier transform infrared (FT-IR) spectrum of SBA-15 was monitored by Bruker Vector 22 spectrometer using potassium bromide (KBr) plate. X-ray diffraction (XRD) was performed on a Bruker D8 Advance powder diffractometer using Ni filtered CuKa radiation (λ=1.54056 Å). The morphology of nanoporous was investigated by a scanning electron microscope (FESEM-TESCAN MIRA3). The surface area, pore-volume, and average pore diameter were evaluated according to the Brunauere Emmette Teller (BET) and Barrette Joyner Halenda (BJH) equations.

***Drug treatments***

A total number of 24 rats were randomly allocated to three groups, including a sham control group, and two HE model groups. The sham control and one group of the HE model rats received 1 ml/kg saline subcutaneously (SC), and the another HE model group treated with SBA-15 (0.2 mg/kg, SC) every 48 hr after the surgery till day 28 of BDL. SBA-15 (0.2 mg/kg) was added to 1 ml saline, sonicated for complete dissolving, and injected subcutaneously according to Hudson *et al*. (2008), which reported that the SC administration of SBA-15 is safer than of both IV and IP routes in rats (38). There are also some reports that the degradation of SBA-15 in simulated body fluid during 24 hr is decreased by increasing the concentration from 0.1 mg/ml with more than 90% degradation to almost 50% degradation for 0.5 mg/ml concentration (39). Therefore, considering the above-cited data and also after a pilot study, we injected SBA-15 at a low dose (0.2 mg/kg, SC) with 48 hr intervals for *in vivo* compatibility, and also to be confident of the complete degradation of the injected drug before the next injection. 

***Behavioral testing***

A locomotion apparatus consisted of a clear Plexiglas container 40 (L) × 40 (W) × 40 (H) cm was used to examine locomotor activity and rearing behavior weekly on days 1, 7, 14, 21 and 28 of BDL. The floor of the apparatus was divided into four equal-sized (20 × 20 cm) squares (Borj Sanat Azma Co, Tehran, Iran). After acclimatization for 30 min, each rat was positioned in the apparatus, and each animal’s behavior was recorded for 5 min by a video camera located 100 cm above the arena floor. The operator was blinded to the groups and treatments. Then, we counted the number of horizontal crossings with all four paws between the squares as an index of locomotion, and the total numbers of rears on hind limbs as an index of rearing behavior. 

***Examining plasma levels of ammonia***

On day 28 of BDL, we anesthetized each animal, collected blood samples freshly via heart puncture, and added the samples to tubes containing ethylenediaminetetraacetic acid (EDTA) for prevention from coagulation. Immediately after the blood sampling, we examined plasma levels of ammonia (Biorexfars Diagnostics, Shiraz, Iran) according to the manufacturer manual (34). 

***Total RNA extraction and cDNA synthesis***

Each rat was decapitated on day 28 of the laparotomy, rat brain was detached from the skull, and the PFC was immediately separated on an ice-cold glass surface for evaluating the Jnk3 and p38 gene expression. We used a Super RNA Extraction Kit to extract total RNAs based on the manufacturer’s protocol (Yekta Tajhiz Azma, Tehran, Iran). We weighed 25 mg of the PFC from each rat and homogenized in 800 µl of a lysis buffer existed in the kit by using a Homogenizer (Heidolph, Germany). We examined the quality of the extracted total RNAs on 1% agarose gel electrophoresis, and assessed their quantity spectrophotometrically (Eppendorf BioPhotometer 6131, Germany). The RNA concentrations were equalized between the biological repeats, and then reverse transcription was carried out based on the instructions of the kit manufacturer (Thermo Fisher scientific, USA).

***Quantitative real-time PCR (qPCR)***

The qPCR method was performed according to our previous published studies (34, 40). First, we examined the expressions of three reference genes including, cyclophilin A (Cycl A), beta-actin (Actb), and glyceraldehyde-3-phosphate dehydrogenase (Gapdh) in both control and intervention groups with a real-time thermal cycler for selecting the best reference gene (iCycler 96, Roche, Germany). According to the results of the NormFinder method in GenEX 6.1 Software (MultiD Analyses AB, Goteborg, Sweden), we selected the Gapdh gene as the internal control gene. Then, quantifications of the Gapdh, p38, and Jnk3 MAPKs genes were performed in independent triplicate reactions (as technical repeats) for each sample. The reaction volume was 20 µl, including 2 µl of a mixture of forward and reverse primers (5 µmol), 5 µl cDNA, 3 µl of ultrapure molecular biology grade ddH_2_O, and 10 µl super SYBR green qPCR master mix 2X (Yekta Tajhiz Azma, Tehran, Iran). The Ct values of the Jnk3 and p38α in the qPCR amplification curves were normalized against the respected Ct values of Gapdh in each experimental group to calculate the ΔCt value, and then the resulting ΔCt values in control group were subtracted from that in the intervention group to calculate the ΔΔCt value for each interested gene. Finally, the 2^-ΔΔCT ^value was used for presenting the fold changes in the gene expression. The primers’ sequences were provided in Table 1.

***Statistical analysis***

We examined normality of the data with the Shapiro–Wilk test. We performed two-way repeated measure ANOVA (RM-ANOVA) for analyzing the behavioral data because of having two factors, including SBA-15 as the first factor with two levels and days of testing as the second factor with five levels. We also used Holm-Sidak *Post hoc* test for pairwise comparisons whenever appropriate. We analyzed the ammonia and qPCR data with the Student t-test because of evaluating the effect of only one factor on day 28 of BDL between two groups. The statistically significant level was set at *P*<0.05. SigmaPlot 12.5 software was used for all statistical analyses and drawing the plots (Systat Software, San Jose, CA). 

## Results

***Characterization of the mesoporous silica SBA-15***

**Figure 1:**
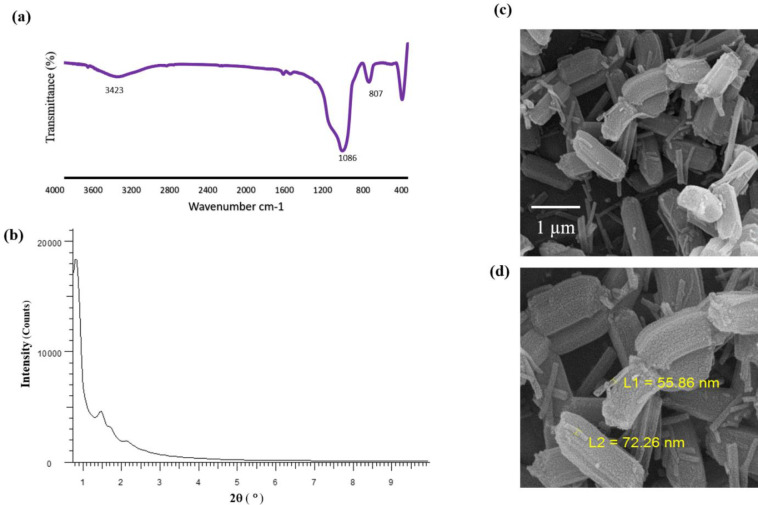
Characterization of mesoporous silica SBA-15. (a) the FT-IR spectra, (b) the XRD patterns, and (c and d) the scanning electron micrographs of the synthesized mesoporous silica SBA-15

In FT-IR spectra of SBA-15, one broadband between 3200 to 3600 cm^-1 ^(SiO-H ), an asymmetric stretching vibration of Si-O-Si in ῡ=1000-1200 cm^-1^, and one symmetrical vibration in ῡ=807 cm^-1 ^were observed (Figure 1a) (41, 42). The XRD pattern of the calcined SBA-15 shows three reflections, including a very intense peak (100) and two additional peaks (110) and (200) with lower intensities (Figure 1b) (43). The results of scanning electron microscopy (figure 1c, d) revealed that SBA-15 nanoparticles have a shape close to the short rod with the friable surface (44-46). BET surface area (845 m^2^/g) and pore size diameter (11.4 nm) of SBA-15 was obtained with BET and BJH nitrogen adsorption-desorption methods (45). 

***The SBA-15 treatments had no significant effect on locomotion in HE model rats throughout 28 days of BDL***

**Figure 2:**
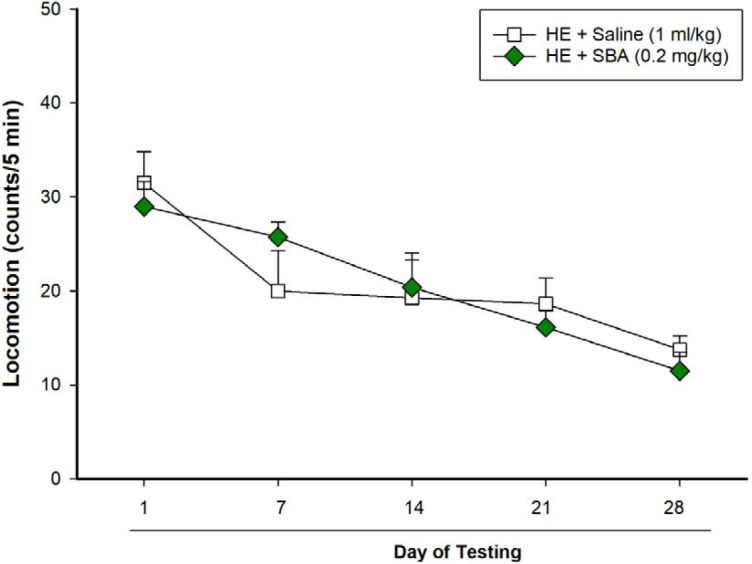
Effect of mesoporous silica SBA-15 on locomotion in rats with BDL. Two groups of rats (n=8) with BDL were used. One group received saline (1 ml/kg), but the other group received SBA-15 (0.2 mg/kg) every 48 hr for 28 days. The rats in both groups were tested for locomotion on days 1, 7, 14, 21, and 28 of BDL. Each point represents the mean±SEM of the total locomotion of eight rats in each group. BDL: Bile duct ligation

The two-way RM-ANOVA indicated no significant interaction among SBA-15 (as the first factor) with days of testing (as the second factor) on locomotion in HE model animals [F (4, 56)=1.37, *P*>0.05]. The results also revealed no significant effect for SBA-15 on locomotion in HE model animals [F (1, 56)=0.0005, *P*>0.05], but a significant effect was obtained for days of testing [F (4, 56)=18.3, *P*<0.001] on locomotion across 28 days of BDL (on days 1, 7, 14, 21 and 28 of BDL), which indicates the impairment of locomotion in HE-model rats during 28 days after BDL (Figure 2). 

***The SBA-15 treatments significantly prevented the impairment of rearing behavior on days 7 and 14 of BDL in HE model rats***

**Figure 3:**
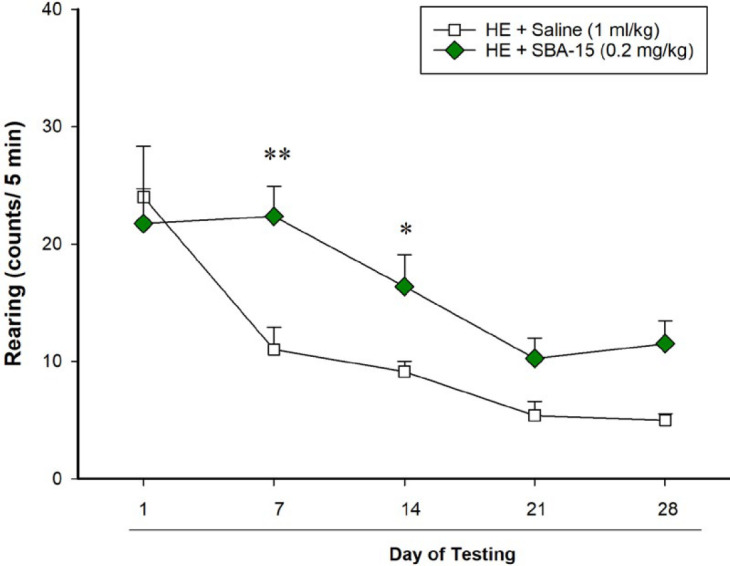
Effect of mesoporous silica SBA-15 on rearing behavior in rats with BDL. The rats in both groups (n=8) that were used for examining locomotion were also tested for rearing behavior on days 1, 7, 14, 21, and 28 of BDL. Each point represents the mean±SEM of the total rearing behavior in each group. * *P*<0.05 and ** *P*<0.01 compared to the group treated with saline on the same day. BDL: Bile duct ligation

The two-way RM-ANOVA indicated that there was a significant interaction between the two factors (SBA-15 and days of testing) on rearing behavior in rats with BDL treated with either saline or SBA-15 [F (4, 56)=3.18, *P*<0.05]. In addition, the analysis revealed significant effects for factor A (SBA-15) [F (1, 56)=6.65, *P*<0.05], and factor B (days of testing) [F (4, 56)=20.38, *P*<0.001] on the number of rearing behavior. Pairwise group comparisons with the *post hoc* test revealed that SBA-15 banned the decrease in the numbers of rearing behavior in HE model rats with significant effects on days 7 and 14 of BDL (Figure 3).

***The SBA-15 treatments in BDL rats significantly decreased hyperammonemia on day 28 of laparotomy***

**Figure 4:**
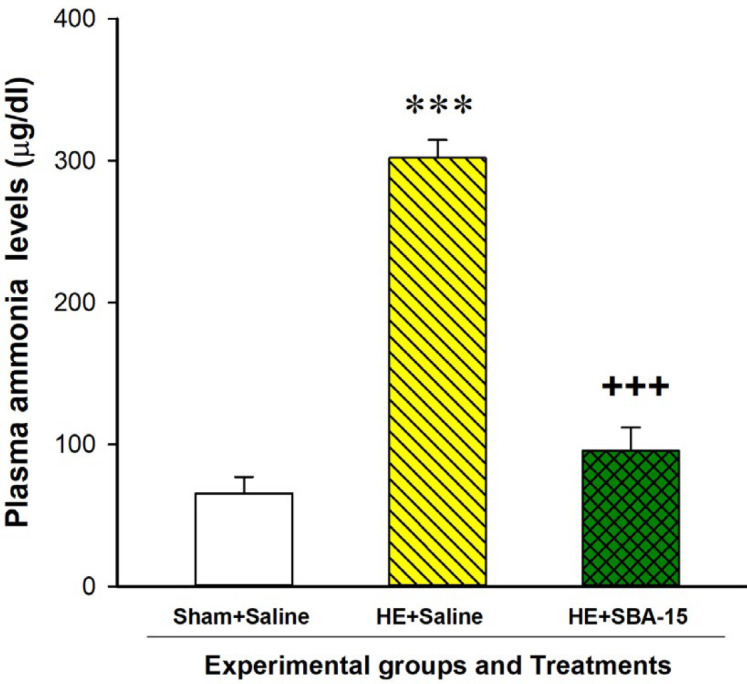
Effect of the mesoporous silica SBA-15 treatments on ammonia levels of plasma. Three groups of rats (n=8) were used. A sham control group and a group of rats with BDL as a HE model group received saline (1 ml/kg), but another HE model group received SBA-15 treatments every 48 hr during 28 days of BDL. On day 28 of BDL, the plasma ammonia levels were examined. Each bar represents the mean±SEM of the plasma ammonia levels of eight rats in each group. *** *P*<0.001 compared to the sham control group. +++ *P*<0.001 compared to the HE model group treated with saline. BDL: Bile duct ligation, HE: Hepatic encephalopathy

We have recently reported substantial increases in the serum levels of bilirubin and hepatic enzymes, as indices of the liver dysfunction, in rats with BDL compared to the control group after 28 days of BDL (34). Analysis of the present results of plasma ammonia levels with the Student t-test also revealed that the BDL rats treated with saline had a significant increase in plasma ammonia level on day 28 of BDL in comparison with the sham control group [t (10)= - 13.8, *P*<0.001]. As the first report, our present results also indicated that the plasma ammonia level in the BDL rats treated with SBA-15 significantly decreased compared to the BDL rats treated with saline on day 28 of BDL [t (10) = 10.02, *P*<0.001] (Figure 4). All of these data confirm that BDL has been completely performed.

***The Jnk3 gene expression in the PFC of HE model animals decreased on day 28 of BDL, which was reinstated by the SBA-15 treatments to a basal level***

**Figure 5:**
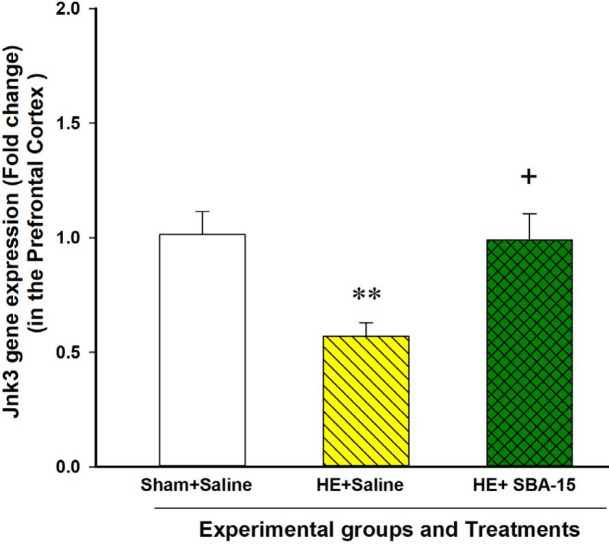
Effect of mesoporous silica SBA-15 on the Jnk3 gene expression in the PFC. Each bar represents the mean±SEM of the Jnk3 gene expression in the specified group. ** *P*<0.01, compared to the sham control group and + *P*<0.05 compared to the HE model group treated with saline. HE: Hepatic encephalopathy, PFC: Prefrontal cortex

Analysis of the qPCR results with the Student t-test showed a significant attenuation in the Jnk3 gene expression in the PFC of HE model rats treated with saline in comparison with the sham control group on day 28 of the schedule [t (6)=3.8, *P*<0.01]. The qPCR results also revealed that the SBA-15 treatments for 28 days in the BDL rats restored the decreased level of the Jnk3 gene expression in the PFC to a level similar to that in the sham control animals treated with saline (Figure 5). 

***The p38α gene expression in the PFC of HE model rats remained with no significant change but increased after the SBA-15 treatments on day 28 of BDL***

**Figure 6:**
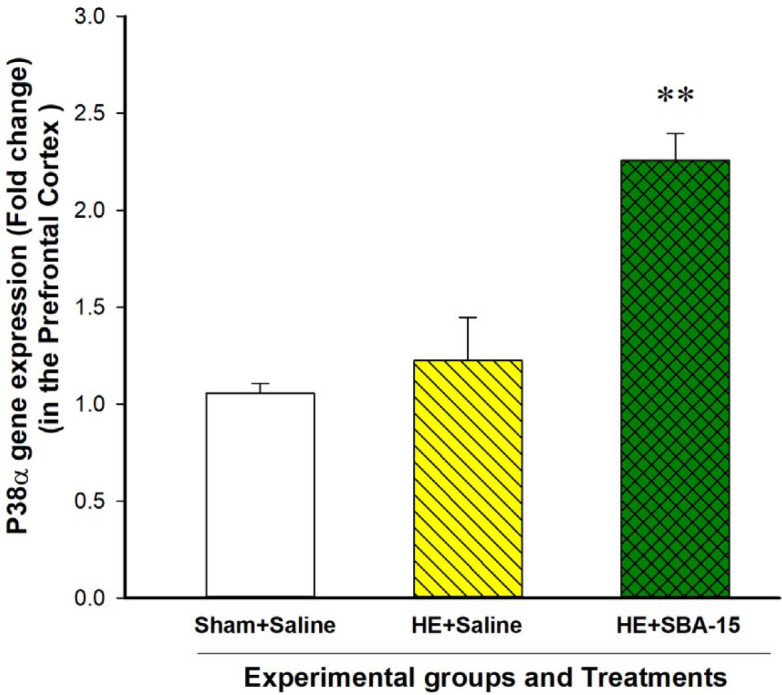
Effect of mesoporous silica SBA-15 on the p38α gene expression in the PFC. Each bar represents the mean±SEM of the p38α gene expression in the specified group. ** *P*<0.01, compared to the HE model group treated with saline. HE: Hepatic encephalopathy, PFC: Prefrontal cortex

Analysis of the qPCR results with the Student t-test indicated no significant difference in the gene expression level of p38α in the PFC on day 28 of BDL between the HE model rats and the sham control group treated with saline [t (6)=-0.7, *P*>0.05]. However, the SBA-15 treatments in the HE model rats significantly increased the p38α gene expression compared to the HE model rats treated with saline on day 28 of BDL [t (6)= -3.9, *P*<0.01] (Figure 6). 

**Table 1:** The sequences of the specific gene primers and the respective amplicon sizes used for real-time PCR

Gene	Sequences (5′-3′)	Amplicon size (bp)
GAPDH	F: AGTGCCAGCCTCGTCTCATA R: GTAACCAGGCGTCCGATAC	77
Actb	F: GCAGGAGTACGATGAGTCCG R: ACGCAGCTCAGTAACAGTCC	74
Cycl A	F: GTTCTTCGACATCACGGCT R: CACGAAAGTTTTCTGCTGTCT	95
P38	F: TGACGAAATGACCGGCTAC R: AGCCCACGGACCAAATATC	104
Jnk3	F: GCTACAAGGAGAACGTGGAC R: CGGAGTTCCTAGCTGCTCTA	132

GAPDH: Glyceraldehyde-3-phosphate dehydrogenase; Actb: Beta actin; Cycl A: Cyclophilin A

## Discussion

We recently reported that locomotion and rearing behaviors are increasingly impaired in rats with BDL as a rat model of HE during 28 days of BDL (34). In rats treated with mesoporous silica SBA-15 for 28 days in this study, we examined locomotor activity and rearing behavior weekly on days 1, 7, 14, 21, and 28 of the treatment period. The results of locomotor activity indicated no significant differences between the two groups either in the presence or absence of the SBA-15 treatments. However, the SBA-15 treatments could prevent the impairment of rearing behavior, at least partly, on days 7 and 14 of BDL in HE model rats. According to the present results, it can be emphasized that SBA-15 not only has no negative effects on locomotion and rearing behavior but also it may have some positive effects on the impairment of locomotion-based behavior such as rearing in rats with BDL. The impairments in rearing behavior and locomotor activity in the HE model animals may result from neurotransmitters disturbances due to BDL, which the SBA-15 treatments could not improve them. Examining the higher doses of SBA-15 and decreasing the intervals between the injections may have an improving effect but it needs more investigations. Although SBA-15 at a dose of 0.2 mg/kg did not improve locomotor activity and movement coordination, but it had no adverse effects as well. In other studies that SBA-15 was used for decreasing glucose levels in diabetic rats, blank SBA-15 did not affect the glucose level (47). It has also been revealed that the secondary structure of serum albumin was not affected by insulin-loaded mesoporous silica particles (48). Collectively, according to the present results and the above-cited data, we propose that SBA-15 is a biocompatible nanostructure for drug delivery in the treatment of HE. 

According to some previous researches, the imbalances in the brain neurotransmission may underlie the impairments in motor and cognitive functions in HE (4, 49). It has also been indicated that the activation of glial cells and neuroinflammation induced by the increases in plasma ammonia levels is involved in the induction of the cognitive impairment in HE (7, 8). Besides, the changes in inflammatory cytokines, free radicals of oxygen, and the subsequent alterations in signaling pathways molecules may underlie the neural disturbances due to the disease (8, 50-53). According to many published pieces of research, the neurological alterations in HE are induced by both hyperammonemia and inflammation like the two sides of a blade (54, 55). Therefore, some researchers believe that the ammonia lowering strategy may be an effective way to reduce the adverse neurological symptoms in HE (50, 56). In the present study, the biochemical analysis of plasma indicated that the plasma ammonia level on day 28 of BDL in the HE model group treated with saline meaningfully increased compared to the sham control group. Interestingly, the results also indicated that the SBA-15 treatments for 28 days after BDL significantly reduced the increased plasma ammonia level on day 28 of BDL. We have previously reported a hyperammonemia condition due to BDL after 21 or 28 days (21, 34), but the decreasing effect of SBA-15 on the increased level of ammonia in HE model animals is reported in the present study for the first time. This result suggests that mesoporous materials can also be used in the captivation of the amine toxins like ammonia from the body fluids. Hydroxyl groups on the surface of SBA-15 may form the hydrogen bonding with ammonia. Measuring the amount of SBA-15 in urine and performing urine analysis could be complementary data for the present results. Therefore, clarifying the exact mechanisms of SBA-15 as an ammonia lowering agent and its possible adverse effects on other factors in the blood needs more investigations. 

MAPKs are signaling molecules downstream to the inflammatory cytokines and neurotransmitter receptors. The family of MAPKs are serine/threonine kinases, which consists of four sub-groups, including JNK, p38, extracellular regulated kinase 1/2 (ERK1/2), and ERK5/Big MAPK (BMK1) (57). The JNK3 sub-group of the JNK group is predominantly expressed in neurons (58). The p38 sub-group involves four isoforms, including α, β, γ, and δ. According to the accumulating evidence, p38 and JNK MAPKs are essential mediators of inflammatory cytokines (25). Besides, it has been indicated that MAPKs have significant roles in the pathogenesis of HE (30). In support of this claim, it has been reported that the inhibitors of p38 and JNK MAPKs signaling pathways have positive effects in the treatment of the diseases of nervous system because of their capacity in reducing the synthesis of pro-inflammatory cytokines and downstream intracellular signaling molecules (22, 25, 27). Also, the improving cognitive effect of a p38 inhibitor in patients with HE has been reported, which suggests the p38 MAPK as a target point in the treatment of HE (59). Considering the above-cited data, we examined the p38α and Jnk3 gene expression in the PFC of HE model rats in the absence and presence of the SBA-15 treatments during 28 days of BDL to investigate its possible effects on neuroinflammation in HE. 

Results of the present study indicated that the Jnk3 gene expression in the PFC of the HE model rats treated with saline was significantly decreased compared to the sham control group. Our present results also indicated that the SBA-15 treatments in another group of the HE model rats restored the Jnk3 gene expression to the basal level. The decrease in the Jnk3 gene expression in the PFC may be a response to an increased level of inflammatory cytokines. Two possibilities can be proposed for the effect of SBA-15 on the Jnk3 gene expression. First, SBA-15 may affect the Jnk3 gene expression indirectly via decreasing the inflammatory cytokines and subsequent neuroinflammation. Second, the SBA-15 treatments may affect the Jnk3 gene expression directly via influencing the mechanisms that regulate gene expression. However, more experiments are needed to clarify the exact mechanism of the action of SBA-15 on the gene expression of Jnk3 MAPK. The results also indicated that the p38α gene expression in the PFC of the HE model group did not significantly alter compared to the sham control group. However, the SBA-15 treatments increased the p38α gene expression compared to the animals treated with saline. The JNK3 and p38α MAPKs are key regulatory molecules downstream to the receptors of inflammatory cytokines as well as neurotransmitters. Therefore, SBA-15 may affect neuroinflammation in HE via influencing the signaling molecules including JNK3 and p38 α at the molecular level. As a limitation of this study, we propose that western blotting analysis could further confirm the gene expression results. Collectively, the gene expression results have revealed a possible effect of SBA-15 on molecular mechanisms involved in HE, which proposes further evaluations in future studies. 

## Conclusion

It can be concluded that mesoporous silica SBA-15 not only did not have impairing effects on locomotor-based behaviors but also had some improving effects on rearing behavior in HE model animals. Also, it is suggested that mesoporous silica SBA-15 can be used as an ammonia lowering agent or as a safe drug delivery system in the HE treatment. However, more experiments must be performed to completely confirm its positive effects and the exact mechanisms involved in its effects on the MAPKs gene expression and neuroinflammation.
